# Numerical Simulation of Short-Arc-Plasma Characteristics in DC Electrofusion Magnesium Furnaces

**DOI:** 10.3390/ma19184025

**Published:** 2026-09-21

**Authors:** Qing Wang, Xuezhi Li, Hang Dong, Baozhen Yang, Pengfei Liu, Chuanhui Dai, Xiang Shen

**Affiliations:** 1 College of Modern Intelligent Manufacturing, School of Mechanical Engineering, Xinjiang University, Urumqi 830017, China; 107552404176@stu.xju.edu.cn (Q.W.); donghangchn@126.com (H.D.); yangbz@xju.edu.cn (B.Y.); 2 Xinjiang Joinworld Co., Ltd., Urumqi 830013, China; cris6789@163.com (P.L.); huihuang2012@126.com (C.D.); jjgfd6587@163.com (X.S.)

**Keywords:** magnesium electrofusion, DC electric arc furnace, arc plasma, magnetohydrodynamics, arc–melt pool interaction

## Abstract

To clarify the heat transfer and flow characteristics of arc plasma in a DC magnesium electrofusion furnace under short-arc conditions, a two-dimensional axisymmetric magnetohydrodynamic (MHD) model was developed. Coupled electromagnetic, thermal, and flow fields were solved using COMSOL Multiphysics 6.3, and the model was validated against Bowman’s free-arc experimental data. Results show that the arc is electromagnetically constricted into a contracted column, with high-temperature and high-velocity regions concentrated near the arc center. Unlike conventional long arcs, the short arc reaches the anode before the jet fully diffuses, causing momentum to be concentrated on the anode surface and generating pronounced pressure peaks. Higher current increases the arc temperature and jet velocity, thereby strengthening the pressure and shear stresses exerted on the molten pool. In contrast, increasing the arc length reduces the arc temperature, flow velocity, and surface forces, weakening both momentum transfer and heat transfer to the molten pool. Overall analysis reveals that arc length has a more significant effect on arc–molten pool interactions than current and is the dominant parameter governing short-arc behavior. These findings provide guidance for optimizing operating conditions and improving energy utilization in magnesium electrofusion furnaces.

## 1. Introduction

The fully enclosed DC arc furnace technology for magnesium smelting was developed from ultra-high-power AC arc furnace technology and is primarily used for the production of electrofused magnesium from magnesite. A distinguishing feature of DC arc furnaces is the use of graphite or self-baked electrodes as the cathode, while the furnace bottom serves simultaneously as the melting crucible and the anode connected to the power source through a conductor [1]. During the metallurgical process, the stable unidirectional current flow in DC arc furnaces provides a more consistent thermal environment than that in AC arc furnaces. Compared with AC arc furnaces, DC arc furnaces offer several advantages, including a more stable and constricted arc, enhanced molten-pool stirring, a more uniform temperature distribution, and reduced erosion of the furnace lining [2]. In this process, the high-temperature arc plasma supplies the thermal energy required to melt highly refractory MgO, while the heat input can be regulated by controlling the arc current. Although numerical simulations of high-temperature plasmas have been successfully applied in fields such as plasma spraying [3], welding [4], and cutting [5], the development of accurate and reliable numerical models for DC arc furnaces remains a significant challenge due to the high operating power of DC arc furnaces, complex experimental conditions, and the limited accessibility of in situ measurements and observations [6]. For example, Qin et al. [7] successfully produced electrofused magnesium using similar DC arc furnace equipment; however, the underlying mechanisms of heat transfer, plasma flow, and arc–molten pool interactions have not yet been systematically clarified. In particular, for arc furnaces used in MgO single-crystal preparation, a comprehensive understanding of arc-plasma convection and the impact intensity of the arc on the molten pool is essential. Such an understanding provides a basis for developing accurate molten-pool models and determining the boundary conditions required for subsequent simulations of MgO single-crystal growth.

Szekely et al. [8] were among the first to perform numerical simulations of plasma jets in electric arc furnaces and obtained the corresponding temperature and flow-field distributions. Subsequently, Alexis et al. [9] and Wang et al. [10] investigated heat-transfer mechanisms and arc-induced shear stresses acting on molten pools using mathematical models of DC arcs. Ramírez and Trápaga [11] further examined the effects of current and arc length on arc characteristics through a dimensionless representation of DC arc behavior. Long et al. [12] numerically investigated the influence of arc current on plasma characteristics and cathode evaporation, demonstrating that current intensity significantly affects plasma temperature and velocity distributions. Pan et al. [13] established a tungsten–arc–workpiece coupled model and revealed the important roles of heat transfer and fluid flow in determining arc behavior and thermal transport characteristics. More recently, Ogino et al. [14] developed a coupled arc–molten metal model and demonstrated that electromagnetic forces and plasma flow strongly affect molten-metal behavior. Furthermore, Hay et al. [15] emphasized that magnetohydrodynamic (MHD) models have become effective tools for investigating heat transfer, fluid flow, and arc–molten pool interactions in DC electric arc furnaces. Li et al. [16] conducted two-dimensional simulations of the arc-column region and demonstrated that current-density distributions strongly influence plasma-flow characteristics. In addition, the experimental velocity measurements reported by Bowman [17] have become widely accepted benchmark data for validating arc-plasma simulations. However, most existing numerical studies have focused on long-arc conditions commonly encountered in steelmaking electric arc furnaces, where the arc jet undergoes substantial diffusion before reaching the molten pool and the arc–molten pool interaction is mainly governed by distributed heat transfer and shear stresses. In contrast, electrofusion magnesium furnaces typically operate with much shorter arc lengths because of the extremely high melting temperature of MgO and the need for concentrated thermal input. Under these conditions, the arc plasma impinges directly on the molten-pool surface before experiencing significant diffusion, resulting in substantially different heat-transfer and momentum-transfer characteristics. Consequently, arc pressure, shear stress, and plasma-jet momentum may play a more important role in governing molten-pool behavior than under conventional long-arc conditions. Despite their industrial importance, the thermal and dynamic characteristics of short arcs in electrofusion magnesium furnaces have received considerably less attention than those of conventional long-arc systems. Jones et al. [18] reported that short arcs can significantly enhance the transfer of heat and momentum to the molten-pool surface. Although Wang et al. [19] developed a three-dimensional steady-state magnetohydrodynamic model of a short-arc DC furnace and investigated the influence of current on molten-pool behavior, studies focusing on arc-plasma characteristics in magnesium electrofusion furnaces remain limited. This study adopts a bottom-anode configuration, in which current flows from the top cathode through the arc and molten pool to the bottom anode, enabling stable short-arc operation and providing a basis for investigating arc heat transfer and arc–molten pool interactions.

This study investigates the arc-plasma behavior in a DC magnesium electrofusion furnace under short-arc operating conditions. Although previous numerical studies have successfully described the coupled electromagnetic, thermal, and flow fields in DC electric arc furnaces, they have mainly focused on long-arc steelmaking furnaces or conventional plasma melting systems [20,21,22]. In contrast, the present model is specifically developed for the short-arc operating regime of a DC electrofusion magnesium furnace, where the plasma jet reaches the molten-pool surface before significant radial diffusion occurs. The novelty of this study is the establishment of a two-dimensional axisymmetric MHD model to clarify the coupled heat and momentum transfer mechanisms under short-arc conditions, and to systematically compare the relative influences of arc current and arc length on arc–molten pool interactions. These results provide a theoretical basis for optimizing operating parameters and improving energy utilization in magnesium electrofusion furnaces.

## 2. Mathematical Model

### 2.1. Model Assumptions

Because of the complexity of arc-plasma phenomena, the following assumptions were adopted in the numerical model:

(1) The arc plasma was assumed to be in local thermodynamic equilibrium (LTE).
(2) The plasma was assumed to be optically thin, and radiation self-absorption was neglected.
(3) The effects of electrode erosion caused by arc discharge and the sheath regions near the electrodes were neglected.
(4) The arc was assumed to be axisymmetric, and a two-dimensional axisymmetric model was employed for the simulations.
(5) The molten-pool surface was assumed to remain flat and at a constant temperature. The arc plasma was treated as an incompressible turbulent flow.
(6) The plasma was assumed to consist of air at atmospheric pressure. Its thermophysical and electrical properties, including density, viscosity, specific heat capacity, thermal conductivity, electrical conductivity, and radiative heat loss, were assumed to be temperature-dependent only.

The above assumptions inevitably introduce certain limitations to the numerical model. The two-dimensional axisymmetric approximation cannot represent asymmetric arc fluctuations or electrode eccentricity, but it is appropriate for describing the mean behavior of a stable DC short arc. Prescribing a constant molten-pool surface temperature neglects the thermal feedback between the plasma and the molten pool, and therefore the predicted heat transfer should be interpreted as the plasma-side thermal behavior. Neglecting the electrode sheath and electrode erosion mainly influences the local current density and temperature distributions in the immediate vicinity of the electrode surfaces, while the incompressible-flow assumption may slightly reduce the prediction accuracy in regions with extremely large density gradients. Nevertheless, these simplifications allow the coupled electromagnetic, thermal, and flow characteristics of the short-arc plasma to be captured efficiently and are consistent with the objective of the present study.

### 2.2. Governing Equations

During the operation of an electric arc furnace, coupled electromagnetic, thermal, fluid-flow, and radiative transport phenomena occur simultaneously. In this study, the arc plasma is modeled using a two-dimensional axisymmetric magnetohydrodynamic (MHD) framework. The governing equations employed to describe the coupled physical fields are summarized below.

The mass conservation equation is expressed as:

(1) $$\frac{1}{r}\frac{\partial }{\partial r}\left(\rho rv_{r}\right)+\frac{\partial \left(\rho v_{z}\right)}{\partial z}=0$$

The axial momentum conservation equation is given by:

(2) $$\frac{1}{r}\frac{\partial }{\partial r}\left(rpv_{r}v_{z}\right)+\frac{\partial }{\partial z}\left(\rho v_{z}^{2}\right)=\frac{\partial }{\partial z}\left(2\mu \frac{\partial v_{z}}{\partial z}\right)+\frac{1}{r}\frac{\partial }{\partial r}\left[r\mu \left(\frac{\partial v_{z}}{\partial r}+\frac{\partial v_{r}}{\partial z}\right)\right]-\frac{\partial p}{\partial z}+j_{r}B_{\theta }$$

The radial momentum conservation equation is given by:

(3) $$\frac{1}{r}\frac{\partial }{\partial r}\left(\rho rv_{r}^{2}\right)+\frac{\partial \left(\rho v_{z}v_{r}\right)}{\partial z}=\frac{1}{r}\frac{\partial }{\partial r}\left(2r\mu \frac{\partial v_{r}}{\partial r}\right)+\frac{\partial }{\partial z}\left[\mu \left(\frac{\partial v_{z}}{\partial r}+\frac{\partial v_{r}}{\partial z}\right)\right]-2\mu \frac{v_{r}}{r^{2}}-\frac{\partial p}{\partial r}-j_{z}B_{\theta }$$

The energy conservation equation is expressed as:

(4) $$\frac{1}{r}\frac{\partial }{\partial r}\left(rpc_{p}v_{r}T\right)+\frac{\partial }{\partial z}\left(\rho c_{p}v_{z}T\right)=\frac{1}{r}\frac{\partial }{\partial r}\left(r\lambda \frac{\partial T}{\partial r}\right)+\frac{\partial }{\partial z}\left(\lambda \frac{\partial T}{\partial z}\right)+\frac{j_{r}^{2}+j_{z}^{2}}{\sigma }+\frac{5k_{b}}{2e}\left(j_{r}\frac{\partial T}{\partial r}+j_{z}\frac{\partial T}{\partial z}\right)-S_{r}$$

where ρ is the density; $v_{r}$ and $v_{z}$ are the radial and axial velocity components, respectively; p is the pressure; $j_{r}$ and $j_{z}$ are the radial and axial components of the current density, respectively; $B_{\theta }$ is the azimuthal component of the self-induced magnetic field; T is the temperature; σ is the electrical conductivity; $S_{r}$ is the radiative heat loss term; e is the elementary charge; $k_{b}$ is the Boltzmann constant; μ is the dynamic viscosity; and $c_{p}$ is the specific heat capacity at constant pressure.

The momentum equation contains the Lorentz force, while the energy equation includes Joule heating and electron enthalpy transport. Since these source terms depend on the electromagnetic field variables, the electromagnetic field equations must be solved simultaneously with the fluid-flow and energy equations. The governing electromagnetic equations are given below.

The current continuity equation is expressed as:

(5) $$\frac{1}{r}\frac{\partial }{\partial r}\left(\sigma r\frac{\partial \varphi }{\partial r}\right)+\frac{\partial }{\partial z}\left(\sigma \frac{\partial \varphi }{\partial z}\right)=0$$

According to Ohm’s law, the current density is given by:

(6) $$j_{r}=-\sigma \frac{\partial \varphi }{\partial r}$$

(7) $$j_{z}=-\sigma \frac{\partial \varphi }{\partial z}$$

According to Ampère’s law, the magnetic field equation can be written as:

(8) $$B_{\theta }=\frac{\mu_{0}}{r}\int_{0}^{r}j_{z}r\;dr$$

where φ is the electric potential, and $\mu_{0}$ is the permeability of free space (μ_0_ = 4π × 10^−7^ H/m).

The standard k–ε turbulence model was employed to simulate the turbulent flow of the arc-plasma jet. The corresponding transport equations for the turbulent kinetic energy (k) and turbulent dissipation rate (ε) are presented below.

The transport equation for the turbulent kinetic energy (k) is given by:

(9) $$\frac{\partial \left(\rho k\right)}{\partial t}+\frac{\partial \left(\rho kv_{i}\right)}{\partial x_{i}}=\frac{\partial }{\partial x_{j}}\left[\alpha_{k}\mu_{\mathrm{eff}}\frac{\partial k}{\partial x_{j}}\right]+G_{k}+G_{b}-\rho \varepsilon -Y_{M}+S_{k}$$

The transport equation for the turbulent dissipation rate (ε) is given by:

(10) $$\frac{\partial \left(\rho \varepsilon \right)}{\partial t}+\frac{\partial \left(\rho \varepsilon v_{i}\right)}{\partial x_{i}}=\frac{\partial }{\partial x_{j}}\left[\alpha_{\varepsilon }\mu_{\mathrm{eff}}\frac{\partial \varepsilon }{\partial x_{j}}\right]+C_{1\varepsilon }\frac{\varepsilon }{k}\left(G_{k}+C_{3\varepsilon }G_{b}\right)-C_{2\varepsilon }\rho \frac{\varepsilon^{2}}{k}+S_{\varepsilon }$$

where $G_{k}\;$ is the generation of turbulent kinetic energy due to mean velocity gradients; $G_{b}$ is the generation of turbulent kinetic energy induced by buoyancy; $Y_{M}$ represents the contribution of compressibility effects to the turbulent dissipation rate and is neglected in the present study because the arc plasma is assumed to be incompressible; $S_{k}$ and $S_{\varepsilon }$ are the source terms of the (k)- and (ε)-equations, respectively; $C_{1\varepsilon }$, $C_{2\varepsilon }$, and $C_{3\varepsilon }$ are empirical model constants; $\alpha_{k}$ and $\alpha_{\varepsilon }$ are the effective Prandtl numbers for the (k)- and (ε)-equations, respectively; and $\mu_{\mathrm{eff}}$ is the effective viscosity.

### 2.3. Computational Domain and Boundary Conditions

**Figure 1:**
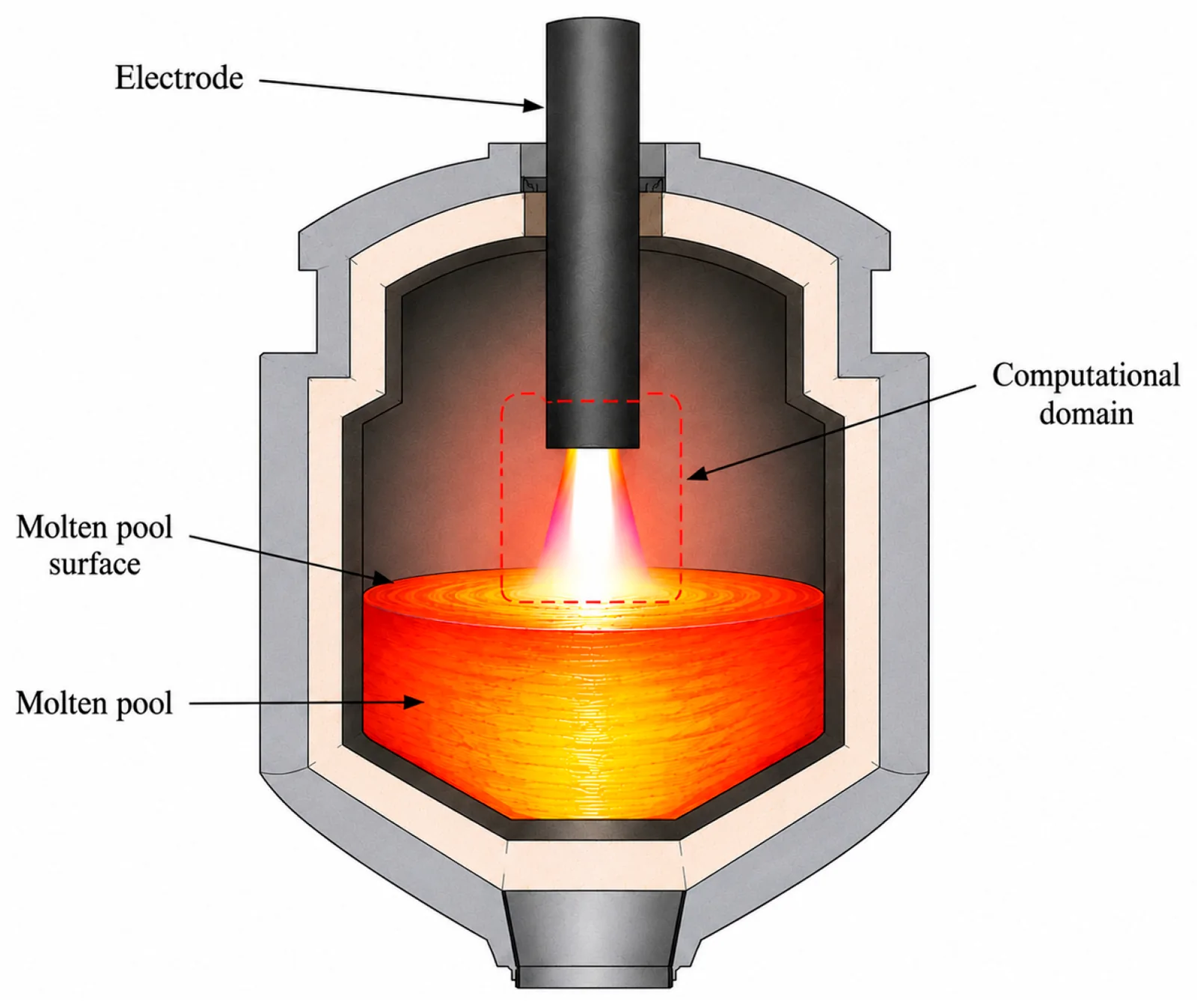
Schematic diagram of the DC electric arc furnace.

Figure 1 illustrates the schematic configuration of the DC arc furnace and the corresponding computational domain. Because the primary objective of this study is to investigate the arc-plasma characteristics, the computational region was restricted to the air domain surrounding the arc column. The molten pool and furnace walls were excluded from the calculation domain because their dimensions are significantly larger than the arc region and their influence on the arc core is negligible. This simplification substantially improves computational efficiency without compromising solution accuracy [23].

**Figure 2:**
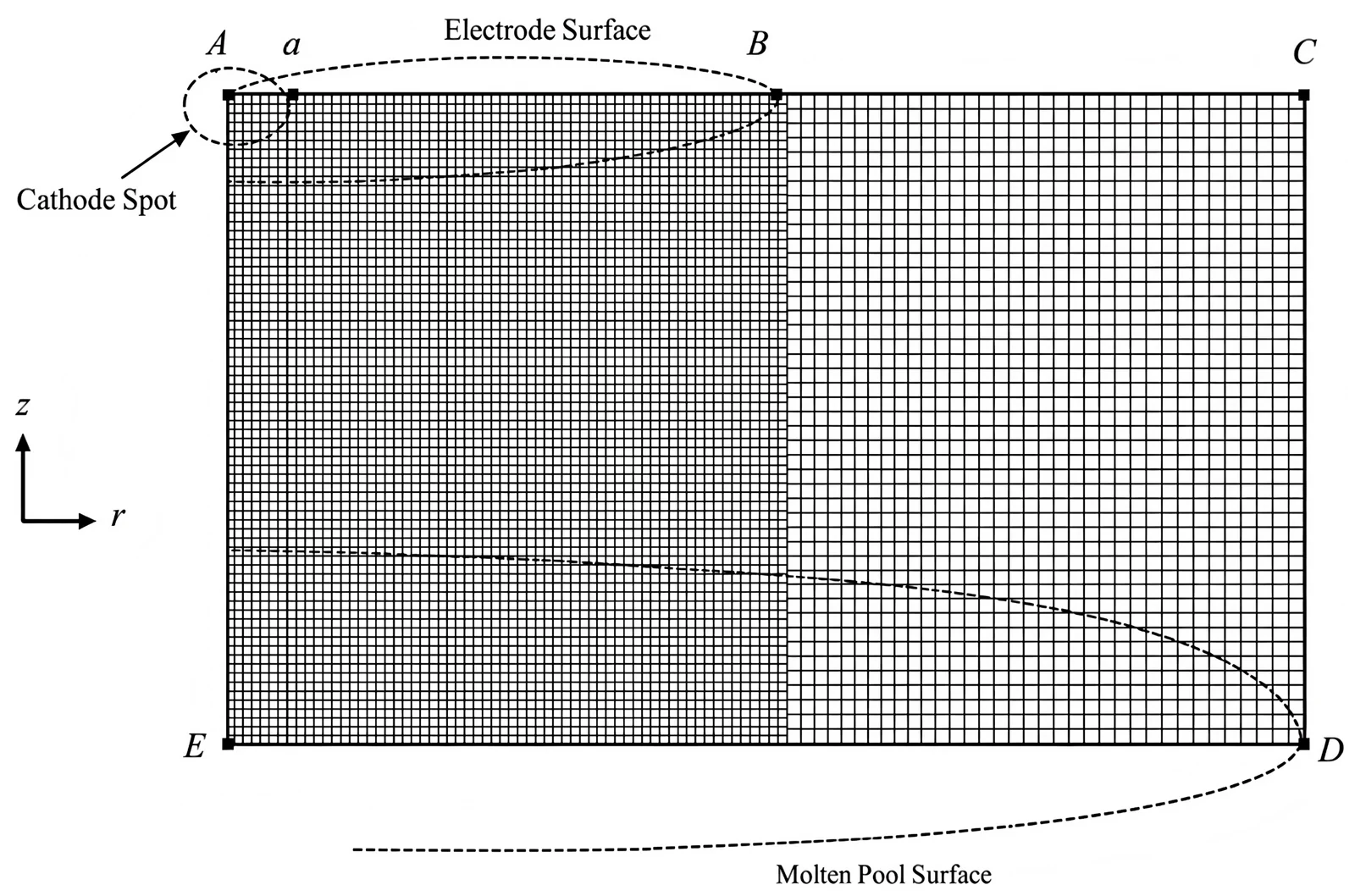
Simplified computational domain of the DC electric arc furnace used for arc-plasma simulation.

As indicated by the highlighted region in Figure 1, the simplified computational domain is presented in Figure 2. To reduce computational complexity, the three-dimensional arc-plasma problem was transformed into a two-dimensional axisymmetric model in the r–z coordinate system. The model was implemented in COMSOL Multiphysics 6.3 (COMSOL AB, Stockholm, Sweden) using a structured mesh. Considering the steep spatial gradients of temperature, velocity, current density, and electromagnetic force in the vicinity of the arc column, local mesh refinement was performed near the electrode tip and arc core region to improve solution accuracy.

A mesh independence study was performed under the reference operating condition of 1000 A and an arc length of 30 mm. Three computational meshes containing 9547, 16,874, and 29,722 elements were employed to evaluate the influence of mesh resolution on the numerical results. The maximum plasma temperature and maximum plasma velocity were selected as the evaluation criteria. As shown in Table 1, the maximum temperature varied from 25,900 K to 25,800 K, while the maximum plasma velocity ranged from 1860 to 1870 m s^−1^. The maximum deviation between the medium and fine meshes was less than 1%, demonstrating that the numerical solution is essentially mesh independent. Therefore, the medium mesh was adopted for all subsequent simulations to achieve a balance between computational accuracy and computational efficiency.

The graphite electrode diameter, arc length, and arc current were set to 5 cm, 3 cm, and 1000 A, respectively. As shown in Figure 2, boundary AB corresponds to the lower surface of the graphite electrode and has a length of 2.5 cm. Boundary AE represents the axis of symmetry and extends over the 3 cm arc region. Since the present study focuses on the arc-plasma behavior, the computational domain was not defined according to the actual furnace dimensions; therefore, boundary AC was located 5 cm from the axis of symmetry to minimize boundary effects on the arc core. Boundary DE represents the interface between the arc plasma and the molten pool. In accordance with the model assumptions, the arc sheath region was neglected. The boundary conditions applied in the numerical model are summarized in Table 2.

Graphite electrodes possess high melting temperatures and emit electrons primarily through thermionic emission [24]. Experimental observations indicate that the cathode spot exhibits limited movement and that the current-density distribution at the cathode is largely independent of the total arc current [25]. Accordingly, cathode-spot motion is neglected in the present model. At the cathode spot (region Aa), the arc current density (J) is prescribed according to the distribution given by:

(11) $$J=2J_{C}\left[1-{\left(\frac{r}{R_{C}}\right)}^{2}\right]$$

where $J_{C}$ is the thermionic electron emission current density, which was set to 3.5 × 10^7^ A/m^2^ according to the Richardson–Dushman thermionic emission model and the cathode boundary condition commonly adopted in previous numerical studies of DC arc furnaces [24,25]. r is the radial coordinate, and $R_{C}\;$ is the radius of the cathode spot.

The relationship between the cathode-spot radius and the thermionic emission current density is given by Equation (12).

(12) $$R_{C}={\left(\frac{I}{\pi J_{C}}\right)}^{\frac{1}{2}}$$

where I is the arc current.

The temperature at the cathode spot (region Aa) was determined according to the Richardson–Dushman equation [24]. The selected current density corresponds to the stable thermionic emission regime of graphite cathodes and has been widely used for high-current DC arc-plasma simulations [24,25]. For a thermionic emission current density of 3.5 × 10^7^ A/m^2^, the cathode-spot temperature was set to 4000 K, which is lower than the melting point of graphite (approximately 4700 K). At the anode boundary (DE), a constant temperature of 3100 K was prescribed to represent the molten MgO surface, corresponding closely to the melting point of MgO (3098 K).

### 2.4. Model Validation

To validate the proposed arc-plasma model, the simulation results were compared with the experimental measurements reported by Bowman [17]. Bowman measured the velocity field of free-burning direct-current arcs with currents up to 2160 A using the well-known steel-ball deflection technique, in which the plasma flow-field distribution was determined from the displacement of a steel sphere combined with theoretical analysis. Owing to the extreme difficulty of obtaining reliable velocity measurements in high-temperature arc plasmas, Bowman’s experimental data have been widely adopted as benchmark data for validating numerical simulations of arc plasmas. To ensure a rigorous comparison, the present model was evaluated under the same operating conditions as those employed in Bowman’s experiments, including a cathode diameter of 50 mm, an arc length of 70 mm, and arc currents of 520 A, 1150 A, and 2160 A.

**Figure 3:**
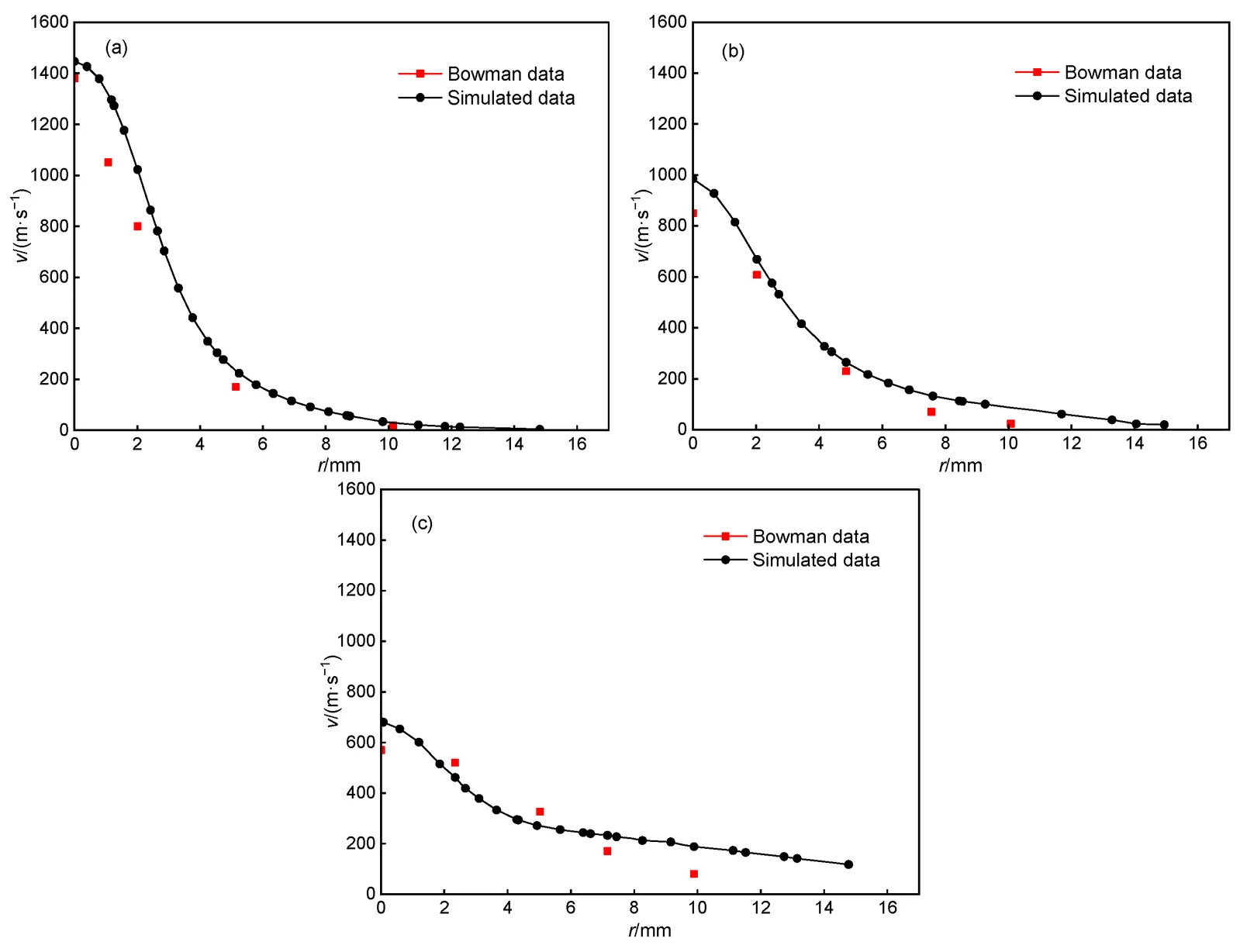
Comparison of the radial distributions of arc-plasma velocity between Bowman’s experimental data and the present simulation at a current of 1150 A: (**a**) 20 mm; (**b**) 38 mm; (**c**) 55 mm from the cathode.

Figure 3 compares the predicted and measured radial velocity distributions at a current of 1150 A for axial distances of 20, 38, and 55 mm from the cathode. The simulation results accurately reproduce the overall shape of the velocity profiles and capture the evolution of the plasma jet along the axial direction. The predicted peak velocities and radial decay trends are in good agreement with the experimental measurements. Furthermore, satisfactory agreement was obtained over the entire range of investigated currents and axial positions. Although minor discrepancies exist in certain regions, these differences are considered acceptable and may be attributed to experimental uncertainties, simplifications introduced in the numerical model, and assumptions regarding local thermodynamic equilibrium. Overall, the deviations between the calculated and measured values remain within an acceptable range, demonstrating that the proposed model can reliably predict the flow characteristics of high-current arc plasmas and providing a solid foundation for the subsequent analysis of temperature, electromagnetic, and flow-field behaviors in DC electrofusion magnesium furnaces.

It should be noted that Bowman’s experimental measurements were obtained from a free-burning DC arc rather than a DC electrofusion magnesium furnace. Consequently, the validation is intended to assess the capability of the numerical model to reproduce the fundamental plasma-flow characteristics instead of duplicating the complete furnace operating environment. Differences in furnace geometry, molten-pool boundary conditions, and thermal conditions may influence the absolute values of the predicted plasma characteristics. However, because both systems are governed by the same coupled magnetohydrodynamic, electromagnetic, and energy transport equations, the Bowman benchmark provides a reliable basis for validating the plasma-flow model prior to its application to short-arc magnesium furnace simulations.

## 3. Results and Discussion

To investigate the influence of operating parameters on arc-plasma behavior, simulations were conducted for arc lengths of 2, 3, and 4 cm and arc currents of 800, 1000, and 1200 A. The resulting flow, temperature, and electromagnetic fields were analyzed to clarify the effects of current and arc length on the characteristics of the DC arc furnace.

### 3.1. Arc-Plasma Characteristics at 1000 A and a 3 cm Arc Length

**Figure 4:**
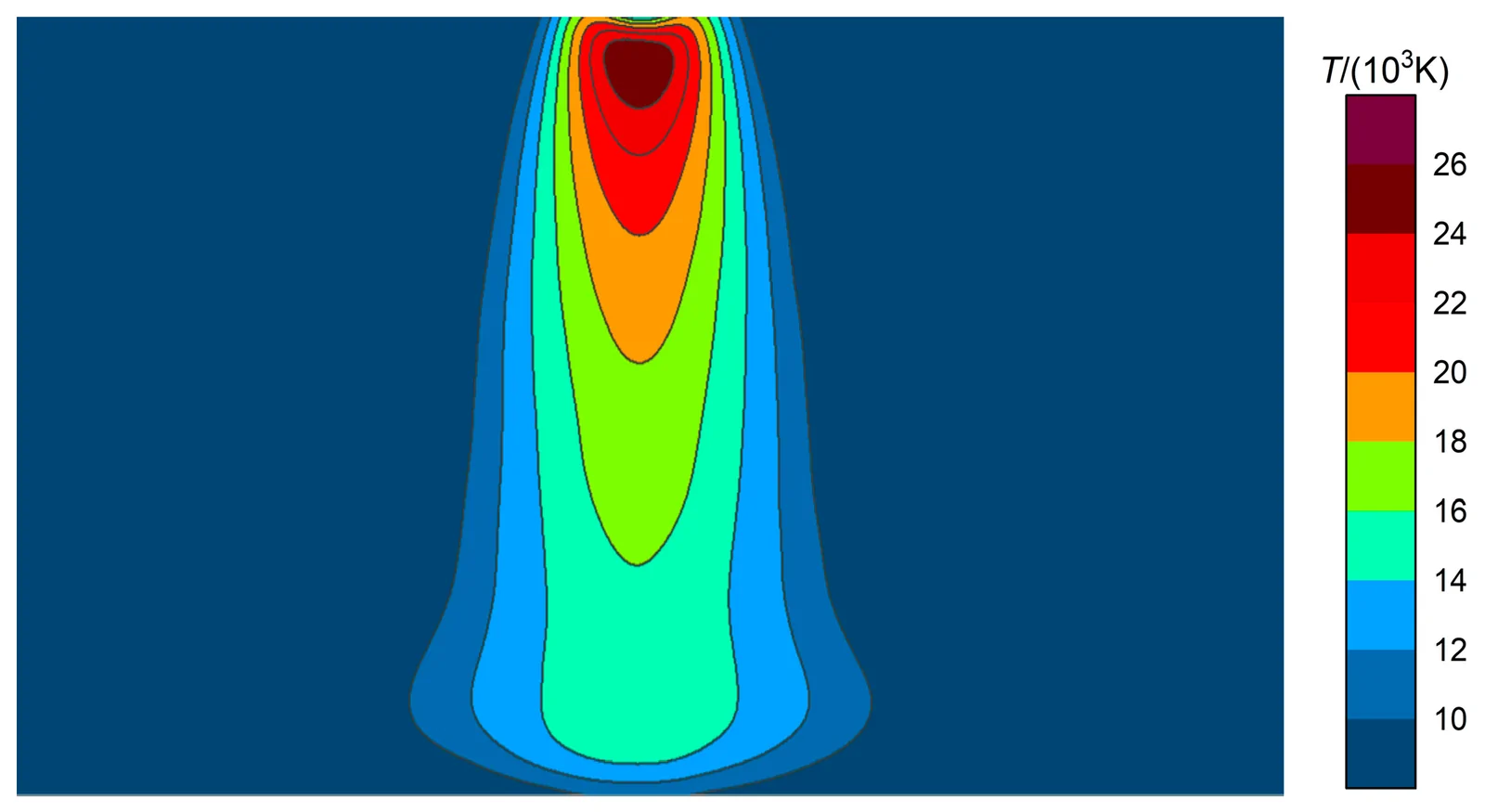
Temperature contours of the arc plasma.

The temperature field of the DC electrofusion magnesium furnace was analyzed under the reference operating condition of a current of 1000 A and an arc length of 30 mm. Figure 4 presents the temperature distribution of the arc plasma. The results show that, under the combined action of turbulent transport and electromagnetic compression induced by the Lorentz force, the arc contracts into a slender column. This temperature distribution is primarily attributed to the high current density near the graphite cathode, where intense Joule heating generates a high-temperature core region. Consequently, the highest temperature occurs near the cathode spot and reaches approximately 25,400 K. The high-temperature region is concentrated along the arc axis, and the temperature gradually decreases in the radial direction owing to heat conduction, turbulent mixing, and radiative heat losses. Meanwhile, the electromagnetic pinching effect suppresses radial expansion of the plasma, further concentrating thermal energy within the arc core. As the plasma flows toward the anode, the current density decreases and energy is continuously dissipated through convection and radiation, resulting in a gradual reduction in temperature. Nevertheless, a relatively large high-temperature region is maintained throughout the arc column, indicating that the short arc can effectively transfer thermal energy to the molten-pool surface (anode boundary). The predicted peak temperature is consistent with the typical temperature range reported for high-current DC arc plasmas in previous numerical studies [10,22,26], indicating that the present model reasonably captures the thermal characteristics of the arc column. Compared with conventional long-arc DC furnaces, the short arc maintains a more concentrated high-temperature region because the plasma reaches the molten-pool surface before significant radial diffusion occurs. This concentrated thermal structure facilitates efficient heat transfer to the molten pool and represents a characteristic feature of short-arc electrofusion magnesium furnaces.

**Figure 5:**
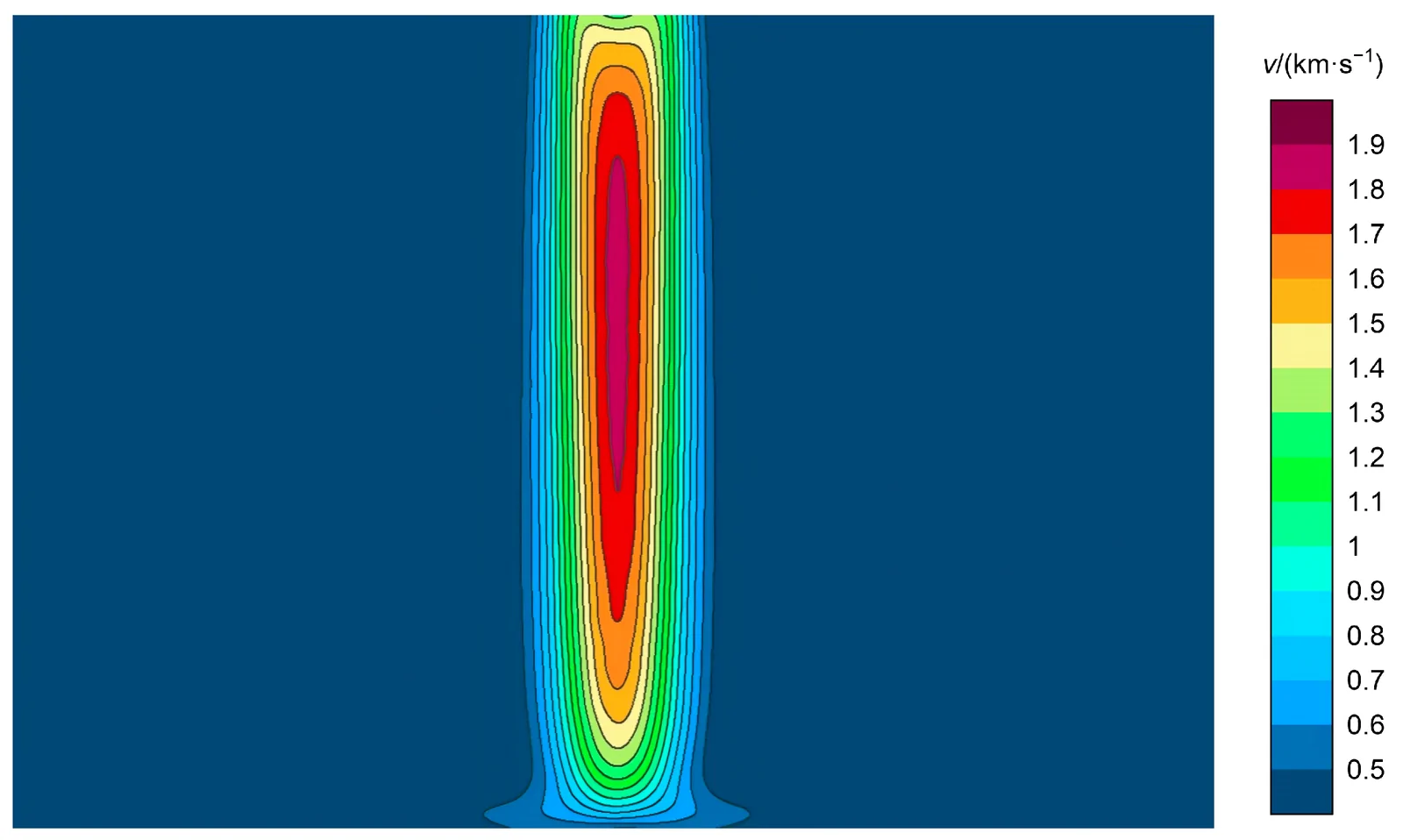
Velocity contours of the arc plasma.

Figure 5 presents the velocity distribution of the arc plasma.

Figure 5 presents the velocity distribution of the arc plasma, which exhibits a typical jet-like structure. The plasma is accelerated near the cathode by electromagnetic forces, and the maximum velocity occurs at the periphery of the cathode spot, reaching approximately 1.87 km s^−1^. A comparison of Figure 4 and Figure 5 shows that the maximum velocity is located slightly downstream of the highest-temperature region. This behavior is attributed to the combined effects of Lorentz-force acceleration and thermal expansion. The elevated temperature near the cathode reduces the plasma density, while the high current density generates a strong electromagnetic driving force, resulting in the continuous acceleration of the plasma jet. Owing to the short arc length, the plasma jet reaches the molten-pool surface (anode boundary) before undergoing significant radial diffusion, allowing a large proportion of its momentum to be transferred directly to the anode region. Consequently, strong momentum exchange enhances molten-pool stirring and promotes heat and mass transfer during electrofusion magnesium smelting. The predicted peak velocity is consistent with the plasma-flow characteristics reported in previous numerical studies and Bowman’s experimental measurements [10,17,22], indicating that the present model reasonably reproduces the dynamic behavior of high-current DC arc plasmas. Compared with conventional long-arc DC furnaces, the short arc maintains a more concentrated high-velocity core, which is a distinctive feature of the short-arc operating regime.

**Figure 6:**
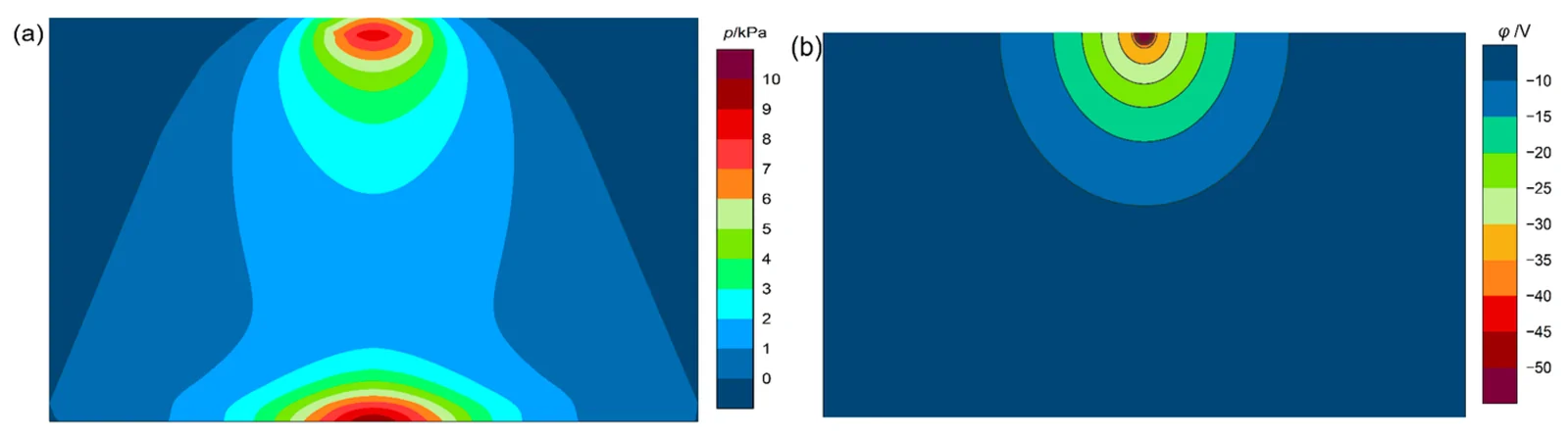
Pressure and electric potential contours: (**a**) pressure field; (**b**) electric potential.

Figure 6 presents the pressure and electric-potential distributions of the arc plasma.

Figure 6a presents the pressure distribution within the computational domain. In the present model, the anode boundary corresponds to the molten-pool surface (Boundary DE); therefore, the pressure discussed below represents the arc impingement pressure acting on the molten pool. Unlike conventional long-arc conditions, where the maximum pressure typically occurs near the cathode, the short arc considered in this study (30 mm) exhibits its maximum pressure at the anode boundary, reaching approximately 9.93 kPa. This behavior is a direct consequence of the short arc length. Under short-arc conditions, the plasma jet impinges on the anode before undergoing sufficient radial expansion, causing a substantial portion of its kinetic energy to be converted into static pressure and producing a pronounced stagnation-pressure peak at the anode surface. This pressure distribution is consistent with the impingement mechanism reported for DC arc plasmas, in which jet momentum is concentrated near the impact region rather than being dissipated through extensive radial diffusion [9,10,22]. As the plasma accelerates downstream from the cathode, the increase in velocity is accompanied by a slight reduction in static pressure within the arc column. Upon approaching the anode, rapid flow deceleration results in a sharp pressure increase, whereas no pronounced negative-pressure region develops near the cathode because of the limited entrainment effect under short-arc conditions. Compared with conventional long-arc DC furnaces, the present short-arc configuration produces a more localized pressure concentration on the anode surface, indicating stronger momentum transfer to the molten pool. This concentrated pressure loading is expected to promote molten-pool deformation and enhance convective heat and mass transfer, highlighting the distinctive arc–molten pool interaction of short-arc magnesium electrofusion furnaces.

Figure 6b presents the electric-potential distribution in the vicinity of the cathode. A steep potential gradient is observed near the cathode spot, giving rise to a strong electric field and consequently a high current density in this region. The resulting concentration of electrical energy produces intense Joule heating, promotes gas ionization, and sustains the formation and stability of the arc plasma. The pronounced potential drop near the cathode indicates that a significant portion of the electrical energy is deposited in the cathode region, which is consistent with the high-temperature zone observed in Figure 4. As the plasma travels toward the anode, the arc column gradually expands and the current density decreases, leading to a continuous reduction in electric potential along the axial direction. This behavior reflects the progressive conversion of electrical energy into thermal energy and kinetic energy during plasma transport. With the anode boundary defined as the zero-potential reference, the maximum potential reaches approximately 49 V. This predicted voltage is consistent with the electrical characteristics reported for high-current DC arc furnaces and plasma melting systems [9,10,22], indicating that the predicted maximum potential of approximately 49 V is within the typical operating range of high-current DC arc systems. Compared with conventional long-arc DC furnaces, the shorter conduction path in the present configuration results in a steeper potential gradient near the cathode, thereby enhancing localized Joule heating and promoting concentrated thermal energy transfer under short-arc operating conditions.

### 3.2. Effect of Arc Current on Arc-Plasma Characteristics

**Figure 7:**
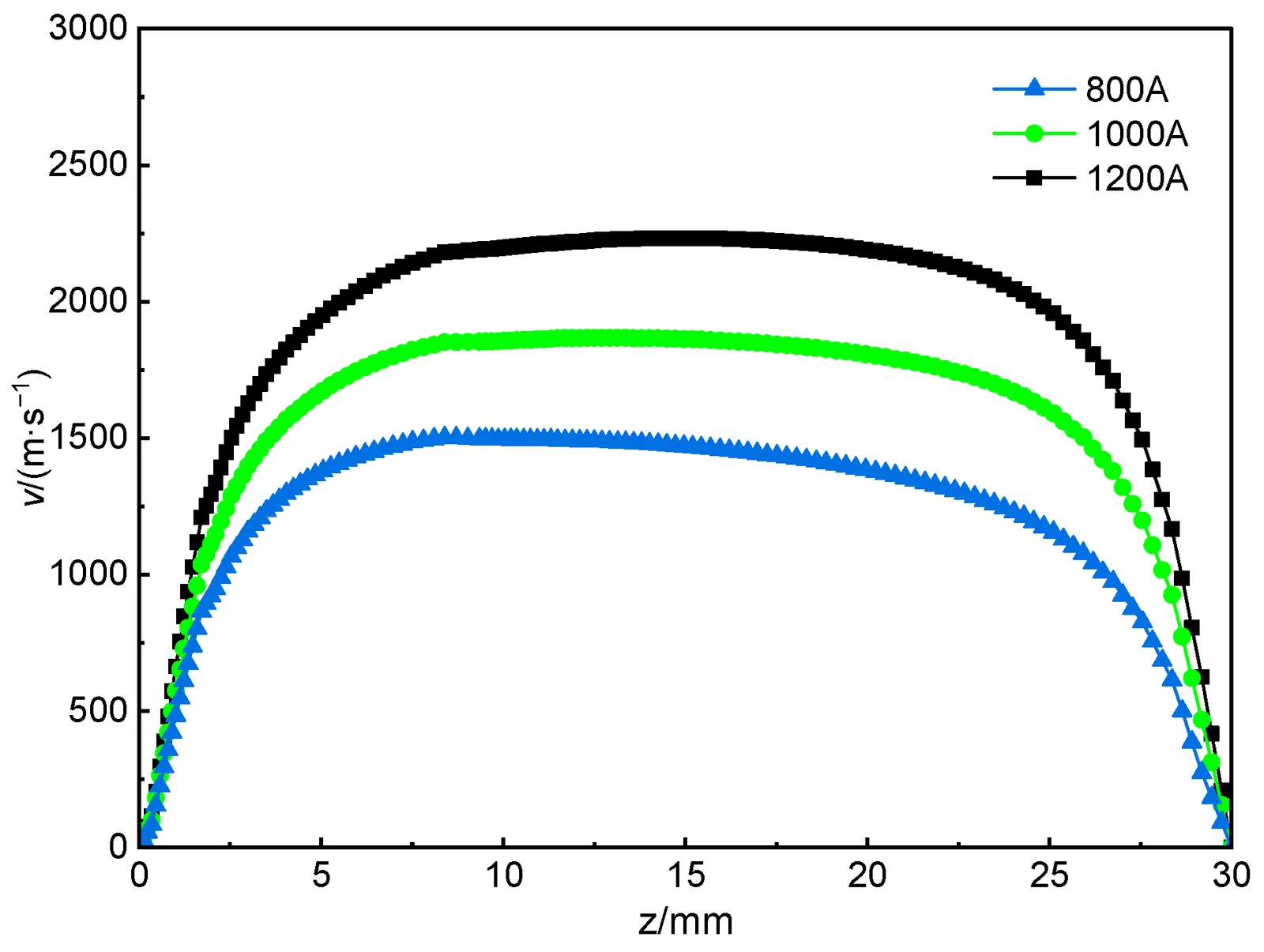
Axial velocity distribution along the arc centerline at different currents.

Figure 7 presents the axial velocity distributions of the arc plasma for arc currents of 800, 1000, and 1200 A. As shown in the figure, the plasma velocity remains relatively low in the vicinity of the cathode spot because of the combined effects of thermal expansion and the electromagnetic forces generated by the high current density. Along the arc centerline, the plasma jet accelerates rapidly within the first 5 mm downstream of the cathode and subsequently decelerates near the anode owing to flow impingement. The maximum plasma velocity increases significantly with increasing current, reaching approximately 1500, 1870, and 2230 m s^−1^ at currents of 800, 1000, and 1200 A, respectively. This trend is primarily attributed to the enhancement of electromagnetic acceleration. Higher currents intensify the Lorentz force acting on the plasma and simultaneously increase Joule heating, thereby promoting gas ionization, thermal expansion, and jet acceleration. A similar increase in plasma velocity with current has been reported in previous numerical studies of high-current DC arc plasmas [10,12,22], demonstrating that electromagnetic driving is the dominant mechanism governing plasma acceleration. Consequently, the stronger plasma jet transfers greater momentum to the anode surface, leading to enhanced arc pressure, increased molten-pool stirring intensity, and improved heat and mass transfer during electrofusion magnesium smelting. These results further indicate that arc current is a key operating parameter controlling the dynamic behavior of short-arc plasma.

**Figure 8:**
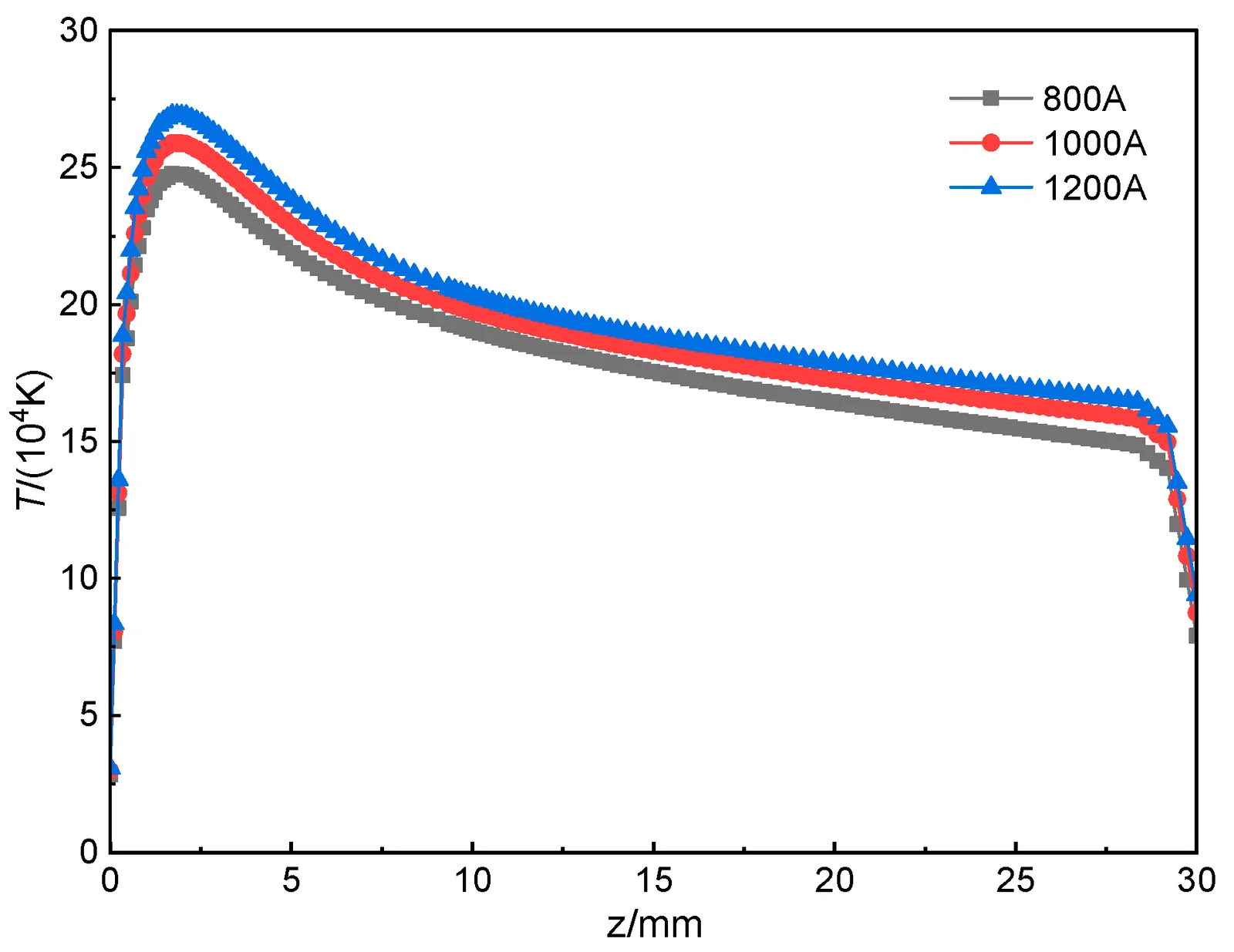
Axial temperature distribution along the arc centerline at different currents.

Figure 8 presents the axial temperature distributions along the arc centerline for arc currents of 800, 1000, and 1200 A. The maximum temperature under all three operating conditions occurs in the vicinity of the cathode surface, reaching approximately 2.43 × 10^4^, 2.54 × 10^4^, and 2.69 × 10^4^ K, respectively. These results indicate that the peak arc temperature increases with increasing current, although the magnitude of the increase is relatively modest. This limited variation is attributed to the expansion of the cathode-spot region, which distributes the additional electrical energy over a larger area and moderates the increase in current density. Moreover, part of the input electrical energy is consumed by plasma ionization, radiation, thermal conduction, and plasma expansion rather than being converted directly into sensible heat. Although the peak temperature changes only moderately, the high-temperature region expands significantly with increasing current, indicating that a larger volume of plasma participates in heat transfer. This trend is consistent with previous numerical investigations of high-current DC arc plasmas, which reported that increasing current primarily enlarges the thermal influence zone rather than producing a proportional increase in peak temperature [10,12,22]. Consequently, higher currents enhance thermal energy transport from the arc to the molten pool and strengthen arc–molten pool interactions. However, increasing the operating current also increases the thermal load on the cathode, suggesting that an appropriate current should be selected to achieve a balance between melting efficiency and electrode service life.

**Figure 9:**
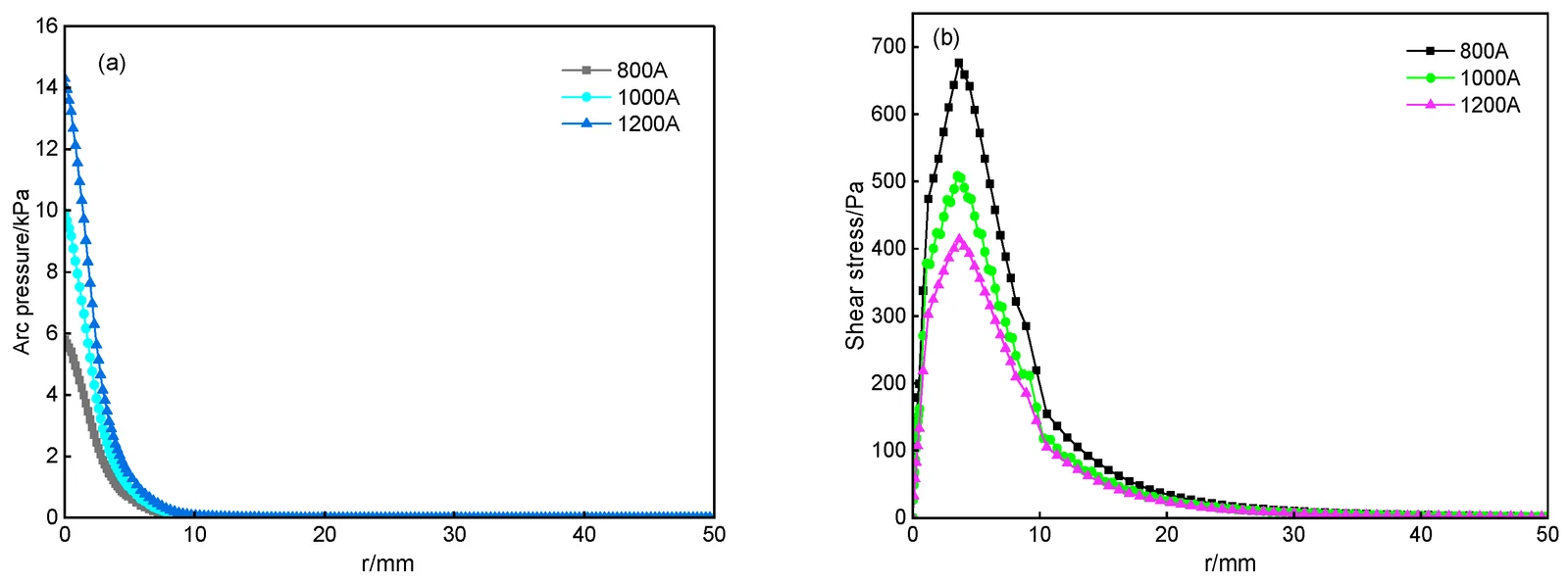
Variations in pressure and shear stress on the anode surface at different currents: (**a**) arc pressure; (**b**) shear stress.

Figure 9a presents the radial distribution of static pressure along the anode boundary for different arc currents. The maximum pressure occurs on the arc centerline and reaches approximately 14,298 Pa at a current of 1200 A. Increasing the arc current significantly increases the peak pressure and slightly enlarges the high-pressure region. This behavior is consistent with the increase in plasma-jet velocity discussed previously, indicating that stronger electromagnetic acceleration produces greater momentum transfer and a higher stagnation pressure at the anode surface. Figure 9b shows the corresponding radial distributions of wall shear stress. The shear stress is zero on the centerline, increases to a maximum value, and then gradually decreases with radial distance. At 1200 A, the maximum shear stress reaches approximately 676 Pa. The simultaneous increase in pressure and shear stress with current agrees well with previous numerical studies of DC arc furnaces, in which higher arc currents enhance both the normal impact force and the tangential momentum transfer of the plasma jet [9,10,22]. The combined analysis of Figure 9a,b demonstrates that increasing the arc current strengthens both the normal pressure and tangential shear stress acting on the anode boundary. As the high-velocity plasma jet impinges on the molten-pool surface, the axial flow is redirected into a radial wall jet, generating substantial shear stresses near the interface. Consequently, higher currents intensify arc–molten pool interactions by enhancing molten-pool circulation and convective heat and mass transfer. However, excessive current also increases the mechanical and thermal loading on the molten-pool surface, indicating that an appropriate operating current is required to balance melting efficiency and process stability.

### 3.3. Effect of Arc Length on Arc-Plasma Characteristics

**Figure 10:**
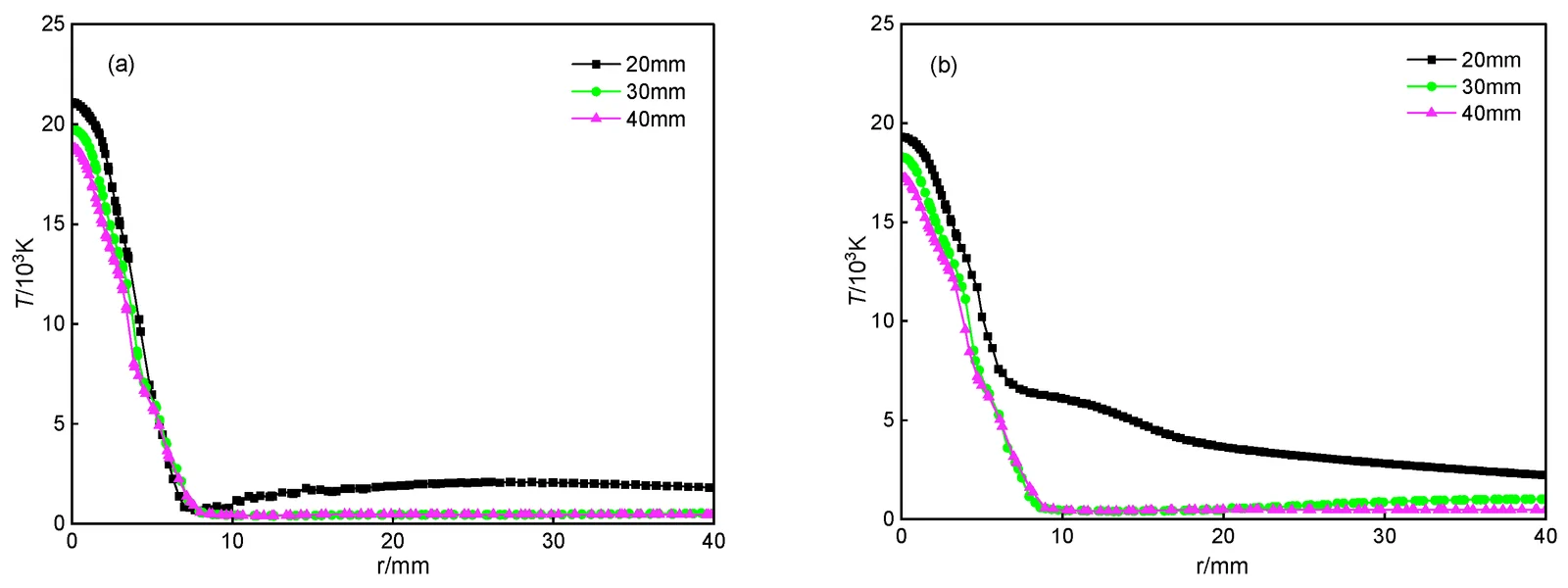
Radial temperature distributions at different arc lengths: (**a**) 1.0 cm below the cathode; (**b**) 1.5 cm below the cathode.

Figure 10 presents the radial temperature distributions at axial distances of 1.0 and 1.5 cm below the cathode for arc lengths of 20, 30, and 40 mm. For all operating conditions, the highest temperature occurs on the arc centerline and decreases with increasing radial distance. At 1.0 cm below the cathode, the peak temperature decreases from approximately 21,090 K to 18,835 K as the arc length increases from 20 to 40 mm, while at 1.5 cm the corresponding values decrease from 19,306 K to 17,310 K. The shorter 20 mm arc maintains a broader high-temperature region than the 30 mm and 40 mm cases, indicating a stronger thermal concentration within the plasma column. These differences are primarily attributed to the increase in arc length. As the conduction path becomes longer, the electrical energy is distributed over a larger volume, resulting in a lower potential gradient, weaker plasma ionization, and greater energy losses through radiation, thermal conduction, and convection. A similar reduction in plasma temperature with increasing arc length has been reported in previous numerical studies of DC arc plasmas [11,22,26], confirming that arc length is a critical parameter governing the thermal characteristics of the arc column. Consequently, increasing the arc length reduces both the thermal intensity and the effective heat-transfer capability of the plasma jet. Compared with conventional long-arc DC furnaces, the short-arc configuration retains a more concentrated high-temperature core, which enhances thermal energy delivery to the molten-pool surface and contributes to improved melting efficiency.

**Figure 11:**
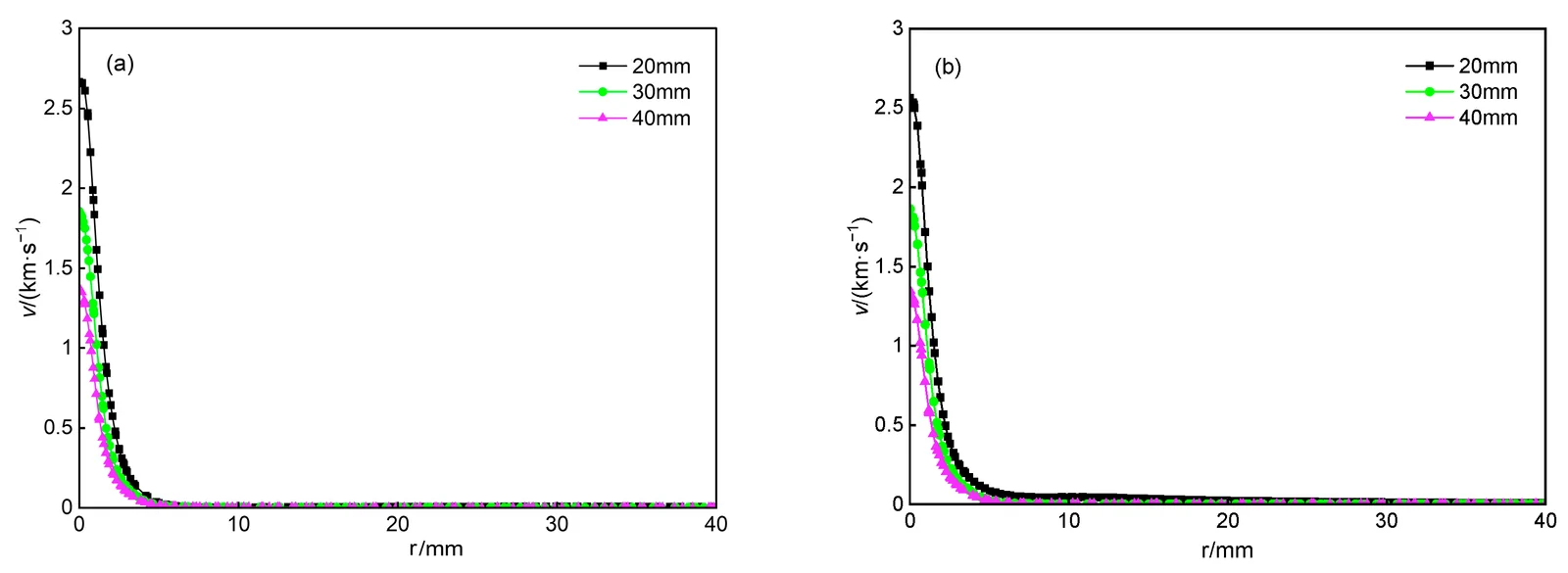
Radial velocity distributions at different arc lengths: (**a**) 1.0 cm below the cathode; (**b**) 1.5 cm below the cathode.

Figure 11 presents the radial velocity distributions at axial distances of 1.0 and 1.5 cm below the cathode for arc lengths of 20, 30, and 40 mm. In all cases, the maximum velocity occurs on the arc centerline and decreases progressively with increasing radial distance. The peak velocity decreases significantly as the arc length increases. At 1.0 cm below the cathode, the maximum velocities for arc lengths of 20, 30, and 40 mm are approximately 2.66, 1.89, and 1.37 km s^−1^, respectively, while the corresponding values at 1.5 cm are 2.56, 1.86, and 1.35 km s^−1^. The shorter 20 mm arc exhibits a larger high-velocity region, indicating stronger plasma-jet acceleration under identical current conditions. The reduction in plasma velocity with increasing arc length is primarily attributed to the decrease in current density and electromagnetic driving force. As the conduction path becomes longer, plasma momentum is distributed over a larger arc column and dissipated through turbulent diffusion and interaction with the surrounding gas, resulting in weaker jet acceleration. This trend is consistent with previous numerical studies of DC arc plasmas, which demonstrated that increasing arc length significantly attenuates plasma-jet velocity and momentum transfer [11,22,26]. Consequently, shorter arc lengths promote stronger momentum transfer and more intense thermal interaction between the plasma jet and the molten-pool surface. Compared with conventional long-arc DC furnaces, the short-arc configuration maintains a concentrated high-velocity core, enabling more efficient momentum delivery to the anode and strengthening arc–molten pool interactions.

**Figure 12:**
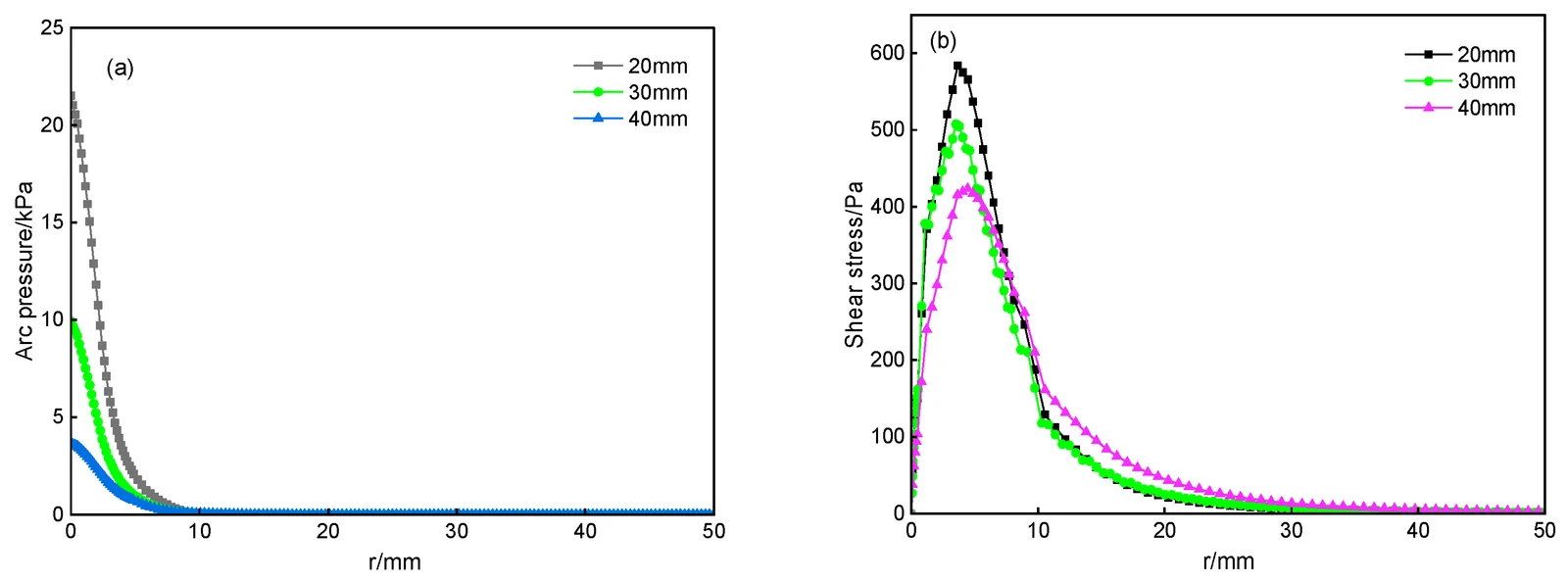
Variations in pressure and shear stress on the anode surface at different arc lengths: (**a**) static pressure; (**b**) shear stress.

Figure 12 presents the radial distributions of static pressure and gas shear stress along the anode surface for different arc lengths. As shown in Figure 12a, the static pressure decreases significantly with increasing arc length under a constant current. The maximum pressure occurs on the arc centerline and reaches approximately 2.1 × 10^4^ Pa at an arc length of 20 mm. A longer arc length weakens electromagnetic acceleration by reducing the electric-field intensity, resulting in lower plasma-jet velocity and a smaller stagnation pressure acting on the anode surface. Figure 12b shows the corresponding radial distributions of gas shear stress. Similar to the pressure distribution, the magnitude of the shear stress decreases as the arc length increases because the plasma jet transfers less momentum to the anode surface. This reduction in both pressure and shear stress is consistent with previous numerical studies of DC arc plasmas, which demonstrated that increasing arc length weakens jet impingement and reduces momentum transfer to the molten surface [11,22,26]. The combined analysis of Figure 12a,b demonstrates that increasing the arc length simultaneously reduces both the normal pressure and tangential shear stress exerted on the anode surface. Compared with current variation, arc length produces a more pronounced influence on arc–molten pool interactions because it simultaneously governs the temperature field, plasma velocity, and effective momentum transfer within the arc column. Consequently, arc length is identified as the dominant operating parameter controlling the thermal and mechanical characteristics of short-arc plasma. Maintaining a relatively short arc length is therefore beneficial for enhancing heat and momentum transfer during electrofusion magnesium smelting.

The present results provide practical guidance for parameter selection in industrial DC electrofusion magnesium furnaces. Under the investigated operating conditions, increasing the arc current enhances plasma momentum and heat transfer, whereas reducing the arc length produces a more pronounced improvement in temperature concentration, jet velocity, anode pressure, and shear stress. These results suggest that maintaining a relatively short arc is generally more effective than simply increasing the current for strengthening arc–molten pool interactions. From an engineering perspective, the operating current should therefore be selected together with the arc length to achieve efficient melting while avoiding excessive thermal and mechanical loading on the molten pool and graphite electrode.

## 4. Conclusions

(1) A two-dimensional axisymmetric magnetohydrodynamic (MHD) model was developed to investigate the arc process in a direct-current bottom-electrode electrofusion magnesium furnace. The electromagnetic, temperature, and flow fields were solved in a fully coupled manner using COMSOL Multiphysics 6.3. Comparison with Bowman’s free-arc experimental data demonstrated good agreement, confirming the reliability of the proposed model.
(2) The results show that the arc plasma exhibits pronounced electromagnetic constriction characteristics under short-arc operating conditions. Owing to the limited arc length, the plasma jet does not fully expand before reaching the anode surface. Consequently, the momentum and thermal energy of the arc are concentrated in the anode region, making the anode surface the primary location of arc impingement and energy transfer.
(3) Increasing the arc current enhances the plasma-jet velocity and enlarges the high-temperature region, leading to higher pressure and shear stress acting on the molten-pool surface. In contrast, increasing the arc length significantly reduces the arc temperature, plasma-jet velocity, pressure, and shear stress, thereby weakening the transfer of heat and momentum from the arc plasma to the molten pool. A comparative analysis shows that arc length has a more pronounced influence on arc characteristics than current variation. Specifically, increasing the arc length from 20 to 40 mm reduced the peak plasma velocity from 2.66 km s^−1^ to 1.37 km s^−1^, corresponding to a decrease of approximately 49%, and decreased the maximum anode pressure by more than 50%. Therefore, arc length is identified as the dominant operating parameter controlling short-arc-plasma behavior and arc–molten pool interactions in electrofusion magnesium furnaces.
(4) The present study clarifies the heat-transfer and flow mechanisms of short arcs in DC bottom-electrode electrofusion magnesium furnaces and provides a theoretical basis for optimizing furnace operating parameters. The findings also establish a foundation for future investigations of molten-pool flow behavior, heat transfer, and single-crystal growth processes in electrofusion magnesium production.

The present study is subject to several limitations. The numerical model is based on a two-dimensional axisymmetric magnetohydrodynamic framework and assumes a prescribed molten-pool surface temperature while neglecting electrode erosion and sheath effects. These simplifications allow the fundamental heat- and momentum-transfer characteristics of short-arc plasma to be investigated efficiently, but they do not fully represent the complex thermo-fluid interactions in industrial electrofusion magnesium furnaces. Future research will focus on establishing a fully coupled arc–molten pool model with dynamic molten-pool flow and heat transfer, providing a more comprehensive basis for process optimization and industrial furnace design.

## Figures and Tables

**Table 1:** Mesh independence study under the reference operating condition (1000 A, 30 mm).

Mesh	Elements	Maximum Temperature (K)	Maximum Velocity (m/s)
Coarse	9547	25,900	1870
Medium	16,874	25,900	1870
Fine	29,722	25,800	1860

**Table 2:** Boundary conditions for the arc-plasma simulation.

Boundary	T/K	φ/V	*A*/(W·h·m^−1^)
AB	4000	$-\sigma \frac{\partial \varphi }{\partial z}=J$	$\frac{\partial A}{\partial n}=0$
BC	3100	$\frac{\partial \varphi }{\partial z}=0$	$\frac{\partial A}{\partial n}=0$
CD	300	$\frac{\partial \varphi }{\partial r}=0$	0
DE	3100	0	$\frac{\partial A}{\partial n}=0$
AE	$\frac{\partial T}{\partial r}=0$	$\frac{\partial \varphi }{\partial r}=0$	$\frac{\partial A}{\partial n}=0$

## Data Availability

The original contributions presented in this study are included in the article. Further inquiries can be directed to the corresponding author.
